# Association between impaired peripheral thyroid hormone sensitivity and coronary heart disease in type 2 diabetes: the potential mediating role of albumin

**DOI:** 10.3389/fendo.2026.1839913

**Published:** 2026-06-17

**Authors:** Binghua Xue, Yalei Liu, Jingyi Wan, Shasha Tang, Xiaoyang Shi, Jin Cao, Huijuan Yuan

**Affiliations:** 1 Department of Endocrinology, Zhengzhou University People’s Hospital, Henan Provincial People’s Hospital, Zhengzhou, China; 2 Department of Nephrology, Division of Blood Purification Center, Zhengzhou University People’s Hospital, Henan Provincial People’s Hospital, Zhengzhou, China

**Keywords:** albumin, coronary heart disease, mediation analysis, thyroid hormone sensitivity, type 2 diabetes mellitus

## Abstract

**Background:**

Type 2 diabetes mellitus (T2DM) is associated with a high risk of cardiovascular mortality, primarily from coronary heart disease (CHD). Thyroid hormone (TH) sensitivity and albumin independently influence cardiovascular health and metabolic homeostasis. However, the potential mediating role of albumin in the association between TH sensitivity and the risk of CHD remains unclear in patients with T2DM.

**Methods:**

This cross-sectional study enrolled 430 hospitalized T2DM patients, categorized into CHD and non-CHD groups. Central TH sensitivity was evaluated using the thyrotrophic thyroxine resistance index (TT4RI), thyroid-stimulating hormone index (TSHI), thyroid feedback quantile index (TFQI), and parametric thyroid feedback quantile index (PTFQI). Peripheral TH sensitivity was estimated by the free triiodothyronine to free thyroxine (FT3/FT4) ratio. Logistic regression, restricted cubic spline and receiver operating characteristic curve were performed. Mediation analysis quantified the indirect effects of serum albumin.

**Results:**

Compared to non-CHD patients, the CHD group exhibited significantly diminished FT3/FT4 ratio and albumin levels. Multivariate logistic regression indicated that a decreased FT3/FT4 ratio was independently associated with CHD, whereas central TH sensitivity indices showed no significant correlation. The FT3/FT4 ratio exhibited the modest discriminative ability for CHD (AUC = 0.740). Furthermore, albumin partially mediated the association between peripheral TH sensitivity and CHD, accounting for 19.3% of the total effect.

**Conclusions:**

Impaired peripheral thyroid hormone sensitivity is positively associated with CHD in T2DM and serum albumin might be one of its biological mechanisms.

## Introduction

Type 2 diabetes mellitus (T2DM) is a metabolic disorder characterized by chronic hyperglycemia, and cardiovascular disease remains the leading cause of mortality in this population (1). Compared with non-diabetic individuals, patients with T2DM have a 2- to 4-fold increased risk of mortality from cardiovascular disease (2, 3). Coronary heart disease (CHD) is a common cardiovascular disorder characterized by inadequate blood and oxygen supply to the myocardium (4). Previous studies have established that diabetes is a well-established risk factor for CHD (5); the 10-year mortality rate for T2DM patients with CHD ranges from 34.5% to 36.4%, posing a significant global health burden (6). Therefore, identifying modifiable risk factors and potential mediators is crucial for optimizing timely interventions and clinical management strategies.

Recent studies have demonstrated that thyroid hormones (TH) are closely associated with cardiovascular diseases (7). Thyroid hormones play a pivotal role in myocardial energy metabolism and remodeling, vascular endothelial function, and lipid metabolism, all of which influence cardiovascular function and susceptibility to CHD (8, 9). Patients with CHD often have abnormal TH levels (10). A meta-analysis revealed that low concentrations of thyroid stimulating hormone (TSH) were associated with a higher risk of all-cause mortality and cardiovascular disease mortality (11). Some researchers have proposed that TH sensitivity provides a superior assessment of the composite effects of thyroid hormones (12, 13), encompassing both central and peripheral sensitivity. Central sensitivity includes the thyrotrophic thyroxine resistance index (TT4RI), thyroid-stimulating hormone index (TSHI), thyroid feedback quantile index (TFQI), and parametric thyroid feedback quantile index (PTFQI) (14). The free triiodothyronine to free thyroxine (FT3/FT4) ratio can estimate the conversion efficiency from FT4 to FT3, representing peripheral sensitivity (15). To date, no study has explored the association between TH sensitivity and risk of CHD in patients with diabetes.

As the predominant multifunctional protein in the systemic circulation, serum albumin possesses significant anti-inflammatory and antioxidant properties (16, 17), and serves as a critical biomarker for cardiovascular risk stratification and prognostic evaluation (18). Recent research has indicated that the serum albumin levels in individuals with CHD are inversely and linearly related to all-cause mortality and cardiovascular death risk (19). In addition, albumin acts as a carrier protein responsible for transporting TH throughout the body (20). However, whether albumin mediates the association between TH sensitivity and CHD in patients with diabetes remains unclear.

Therefore, we investigated the association between TH sensitivity and CHD in patients with T2DM, hypothesizing that serum albumin may be associated with this relationship and potentially act as a mediator.

## Methods

### Participants

From May 2019 to May 2025, 430 patients with T2DM hospitalized in the Endocrinology Department of Henan Provincial People’s Hospital were enrolled in this study. The clinical diagnostic criteria for T2DM and CHD remained unchanged and were strictly followed the diagnostic criteria of the International Society of Cardiology and the World Health Organization (WHO) throughout the study. The specific diagnostic criteria of detailed inclusion and exclusion protocols are described in the Supplementary Methods.

Ethical approval was obtained from the Ethics Committee of Henan Provincial People’s Hospital [NO. 2018(48)]. All procedures were conducted in accordance with the Declaration of Helsinki, and written informed consent was obtained from all participants. All participants underwent an overnight fast of no less than 8 hours. Both fecal and serum specimens from all participants were harvested and preserved at −80 °C pending DNA extraction or sequencing.

### Physical examination and laboratory measurements

All data were obtained from medical electronic records of the study participants. The general information of each patient, including sex, age, body mass index (BMI), history of diabetes and thyroid treatment was collected. BMI (kg/m^2^) = weight (kg) / height squared (m^2^). All laboratory assays were performed at the same central clinical laboratory of Henan Provincial People’s Hospital using identical analytical platforms and standardized reagents. The institutional reference intervals for all parameters remained stable during the study period. Biochemical parameters, including creatinine (Cr), uric acid (UA), total cholesterol (TC), triglyceride (TG), low-density lipoprotein cholesterol (LDL-C), high-density lipoprotein cholesterol (HDL-C), alanine aminotransferase (ALT), aspartate aminotransferase (AST) and serum albumin (ALB) were measured using an automated biochemical immunoassay workstation (Aeroset, Abbott Laboratories, USA). Plasma glycated hemoglobin (HbA1c) concentrations were measured by automatic high-performance liquid glycated hemoglobin analyzer (H9, Lifotronic Co., China). Serum FT3, FT4, TSH levels were determined with the Cobas e602 analyzer (Roche Diagnostics, Basel, Switzerland). The institutional reference intervals for thyroid function were: FT3 (3.1~6.8 pmol/L), FT4 (12~22 pmol/L), TSH (0.27~4.2 mIU/L).

### Indices of TH sensitivity

Four indices were calculated to assess central TH sensitivity. TT4RI was calculated as FT4 (pmol/L) × TSH (mIU/L) (21). TSHI was calculated as lnTSH (mIU/mL) + 0.1345 × FT4 (pmol/L) (22). TFQI was calculated using the empirical cumulative distribution function as follows: cdfFT4 − (1 − cdfTSH) (14). PTFQI was calculated as Φ((FT4 − μFT4)/σFT4) − (1 − Φ((ln TSH − μlnTSH)/σlnTSH)) (23), where μFT4 = 16.161, σFT4 = 2.131, μlnTSH = 0.688, and σlnTSH = 0.457 (24) for this current Chinese population.

Higher values of TSHI and TT4RI indicate lower impairment in central TH sensitivity. The TFQI and PTFQI range from -1 to 1, negative values indicate higher pituitary sensitivity to TH, whereas positive values indicate lower central sensitivity. Additionally, a lower FT3/FT4 ratio reflects decreased peripheral TH sensitivity (25, 26).

### Statistical analysis

All statistical analyses were performed using SPSS Version 27.0 (IBM, New York, USA) and R software Version 4.5.2 (R Foundation for Statistical Computing, Vienna, Austria). Continuous variables adhering to a normal distribution are expressed as mean ± standard deviation (SD), while non-normally distributed data are presented as medians with interquartile ranges. For two-group comparisons, Student’s *t*-test was applied to normally distributed continuous variables, whereas the Mann-Whitney *U* test was utilized for non-normally distributed data. Categorical variables were analyzed using the chi-square test. Correlations were evaluated using Spearman’s rank test. Multivariable logistic regression models were constructed to identify associated factors, with results presented as odds ratios (ORs) and 95% confidence intervals (CIs). We standardized the FT3/FT4 ratio using Z-scores to entry into the logistic regression models. Receiver operating characteristic curves (ROC) were plotted and the area under the curve (AUC) were calculated to assess model performance. Furthermore, mediation analysis was conducted using the “Mediation” package. A two‐tailed *P* value < 0.05 was considered statistically significant.

## Results

### Characteristics

A total of 430 patients with T2DM were divided into the non-CHD and the CHD groups. The clinical characteristics of all these participants were shown in **Table 1**. There were no significant differences in sex, BMI, FT4, TSH, UA, TT4RI, TSHI, TFQI and PTFQI between the two groups (*P* > 0.05). Compared with the non-CHD group, the levels of FT3, FPG, HbA1c, ALT, AST, ALB, TC, TG, HDL, LDL and FT3/FT4 ratio were lower, while their age, duration and Cr were higher (all *P* < 0.05).

**Table 1:** The Clinical and anthropometric characteristics of T2DM.

Characteristics	non-CHD group (n = 275)	CHD group (n = 155)	*P* value
Sex (M/F)	174/101	105/50	0.351
Age	58.00 (50.00, 63.00)	60.00 (55.00, 68.00)	<0.001
Duration (years)	9.00 (4.00, 16.00)	15.00 (8.00, 20.00)	<0.001
BMI (kg/m^2^)	25.39 (23.31, 28.10)	25.81 (23.59, 27.84)	0.819
FT3 (pmol/L)	4.43 (3.97, 4.89)	4.16 (3.67, 4.59)	<0.001
FT4 (pmol/L)	16.30 (14.88, 18.09)	16.28 (14.75, 17.42)	0.179
TSH (μIU/mL)	1.93 (1.38, 2.90)	2.02 (1.54, 2.82)	0.288
FPG (mmol/L)	7.89 (6.30, 10.58)	7.20 (5.52, 9.50)	0.024
HbA1c (%)	8.20 (7.00, 10.00)	7.65 (6.80, 9.00)	0.002
ALT (U/L)	21.00 (15.80, 31.30)	16.50 (12.30, 25.00)	<0.001
AST (U/L)	19.30 (15.50, 25.20)	17.30 (13.50, 22.00)	<0.001
ALB (g/L)	42.75 (38.64, 45.35)	40.23 (36.84, 43.35)	<0.001
Cr (μmol/L)	62.70 (51.80, 84.00)	73.80 (58.70, 104.50)	<0.001
UA (μmol/L)	323.20 (262.3, 393.00)	337.85 (270.40, 402.50)	0.390
TC (mmol/L)	4.76 (3.93, 5.68)	3.95 (3.19, 4.79)	<0.001
TG (mmol/L)	1.82 (1.22, 2.77)	1.50 (1.14, 2.30)	0.020
HDL (mmol/L)	2.15 (1.28, 2.97)	1.71 (1.15, 2.36)	0.001
LDL (mmol/L)	1.16 (0.93, 1.69)	1.04 (0.83, 1.37)	<0.001
FT3/FT4	0.27 (0.25, 0.30)	0.26 (0.23, 0.29)	0.016
TT4RI	31.96 (23.10, 45.57)	34.28 (25.02, 46.62)	0.394
TSHI	2.91 (2.53, 3.22)	2.95 (2.57, 3.27)	0.692
TFQI	-0.11 ± 0.36	-0.12 ± 0.36	0.915
PTFQI	0.02 ± 0.38	0.02 ± 0.38	0.843

Data are expressed as the mean ± standard deviations or medians (P25, P75) or numbers (%).

BMI, body mass index; FT3, free triiodothyronine; FT4, free thyroxine; TSH, thyroid stimulating hormone; Cr, creatinine; UA, uric acid; TC, total cholesterol; TG, triglyceride; HDL-C, high-density lipoprotein cholesterol; LDL-C, low-density lipoprotein cholesterol; ALT, alanine aminotransferase; AST, aspartate aminotransferase; ALB, serum albumin; TT4RI, thyrotrophic thyroxine resistance index; TSHI, thyroid-stimulating hormone index; TFQI, thyroid feedback quantile index; PTFQI, parametric thyroid feedback quantile index; FT3/FT4 ratio, free triiodothyronine to free thyroxine ratio; CHD, coronary heart disease.

### Association between TH sensitivity and CHD risk

Logistic regression analysis was performed to examine the association between TH sensitivity indices and CHD (**Table 2**). Model 1 was the crude model, while Model 2 was adjusted for age, sex, duration, BMI, Cr, HbA1c, ALT, TC, TG, HDL and LDL. In the unadjusted model, our results showed that FT3/FT4 ratio was inversely associated with the risk of CHD (OR = 0.768, 95%CI 0.624 - 0.944, *P <* 0.05). After controlling for multiple covariates, a lower FT3/FT4 ratio was independently associated with CHD. Furthermore, after stratification by sex, FT3/FT4 ratio was only inversely associated with CHD in females (**Supplementary Table 1**).

**Table 2:** Logistic regression analysis of TH sensitivity and the risk of CHD.

Variables	Model 1			Model 2		
	OR	95%CI	*P* value	OR	95%CI	*P* value
FT3/FT4	0.768	0.624 - 0.944	0.012	0.769	0.610 - 0.971	0.028
TT4RI	1.003	0.990 - 1.016	0.649	1.000	0.986 - 1.015	0.958
TSHI	0.975	0.682 - 1.395	0.891	0.988	0.665 - 1.469	0.953
TFQI	0.901	0.519 - 1.561	0.709	1.055	0.572 - 1.945	0.865
PTFQI	0.962	0.574 - 1.613	0.884	1.185	0.665 – 2.114	0.565

Model 1: crude model, Model 2: adjusted for age, sex, duration, BMI, Cr, HbA1c, ALT, TC, TG, HDL and LDL.

OR, Odd ratio; 95% CI, 95% confidence interval; FT3, free triiodothyronine; FT4, free thyroxine; TT4RI, thyrotrophic thyroxine resistance index; TSHI, thyroid-stimulating hormone index; TFQI, thyroid feedback quantile index; PTFQI, parametric thyroid feedback quantile index; FT3/FT4 ratio, free triiodothyronine to free thyroxine ratio.

### Impaired TH sensitivity and T2DM with CHD

**Figure 1:**
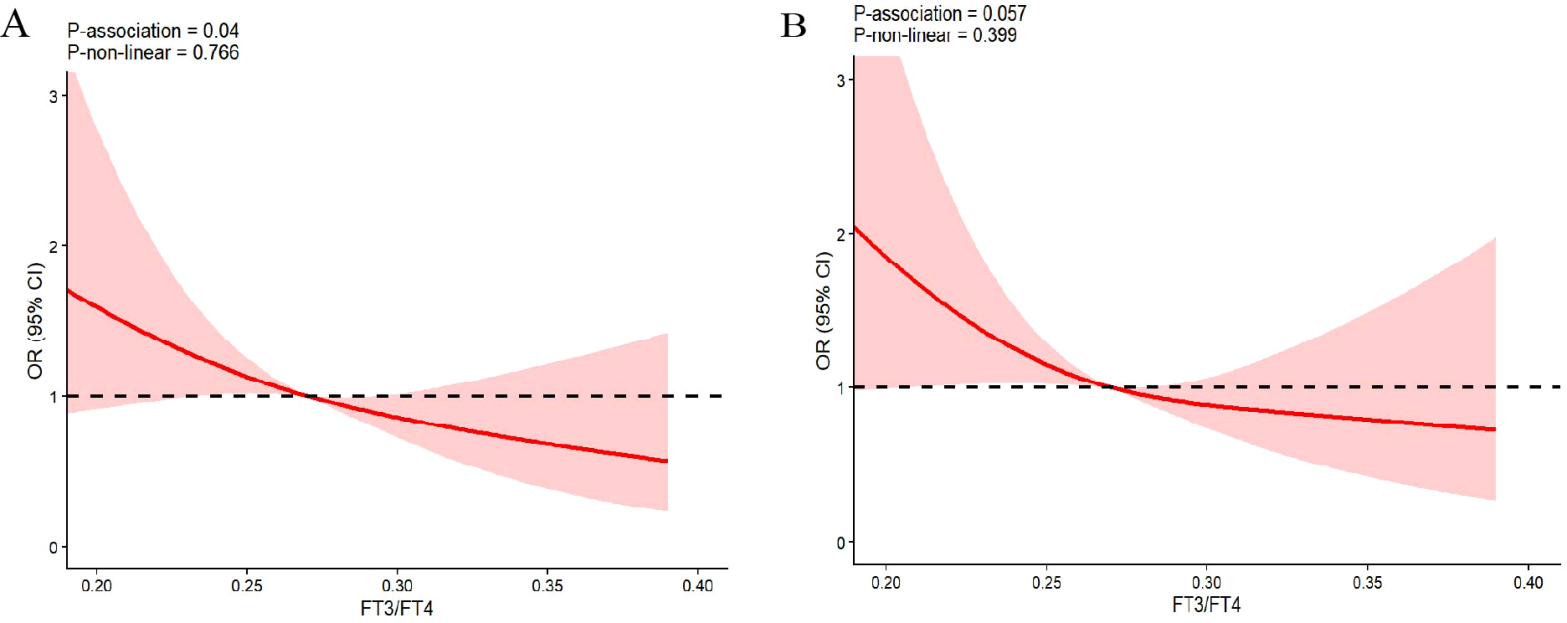
Restricted cubic spline (RCS) analysis for the association between TH sensitivity indices and type 2 diabetes with CHD. **(A)** Crude model, **(B)** Models evaluating FT3/FT4 were adjusted for age, sex, duration, BMI, Cr, HbA1c, ALT, TC, TG, HDL and LDL. FT3/FT4 ratio: free triiodothyronine to free thyroxine ratio.

Restricted cubic spline (RCS) models were employed to evaluate potential nonlinear associations between TH sensitivity and the risk of CHD in patients with T2DM. As shown in **Figure 1A**, the risk of CHD increased with a decreasing FT3/FT4 ratio (*P*_overall_ = 0.040), and this relationship was demonstrated to be linear (*P*_non -linear_ = 0.766). In the adjusted models, we observed a consistent and significant negative correlation trend between the FT3/FT4 ratio and the prevalence of CHD (**Figure 1B**). The RCS curves for central sensitivity indices versus CHD risk are presented in **Supplementary Figure 1**.

### ROC curve of thyroid parameters for predicting T2DM with CHD

**Figure 2:**
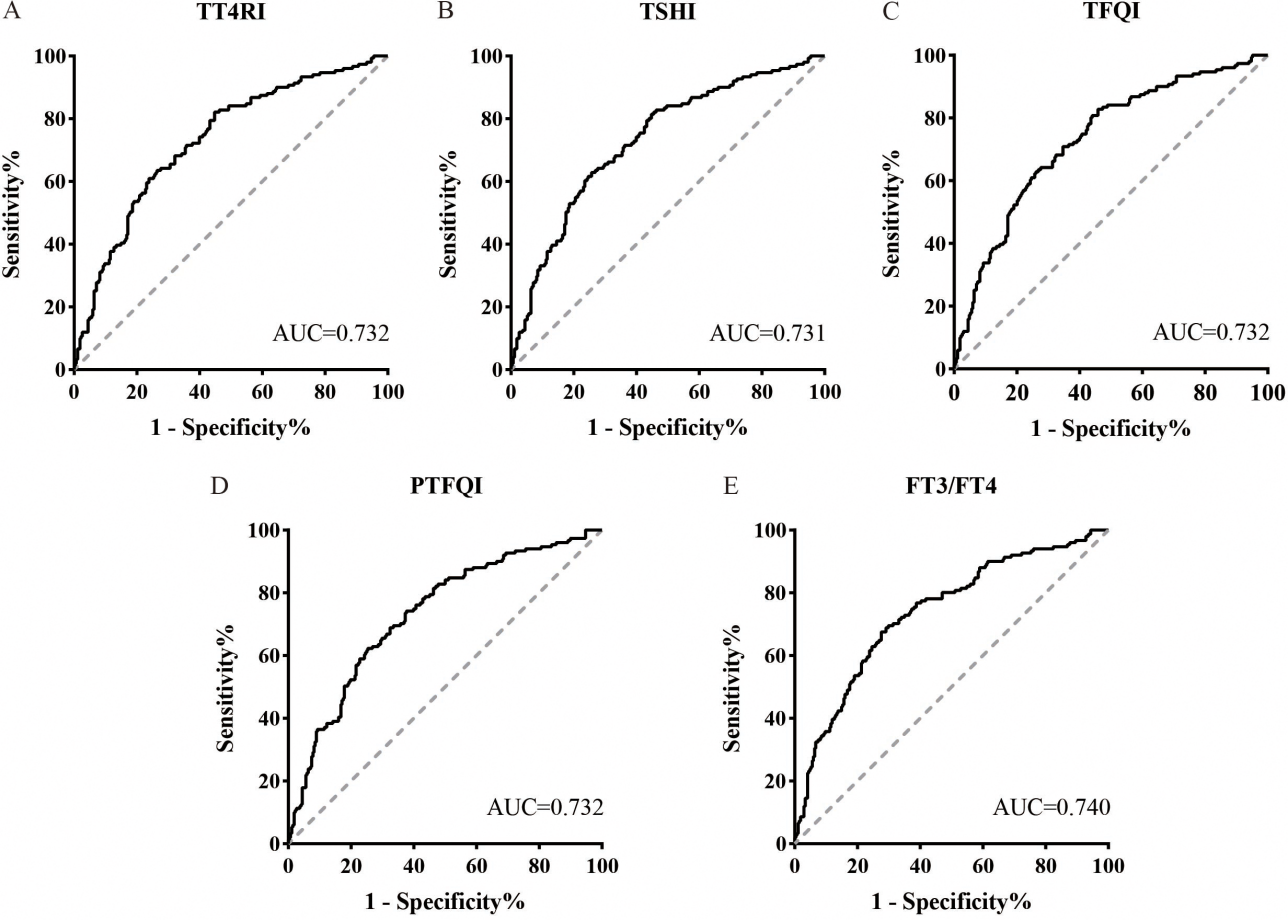
Receiver operating characteristic curve (ROC) of the TFQI, PTFQI, TSHI, TT4RI, FT3/FT4 and CHD risk in patients with T2DM. Models evaluating TH sensitivity were adjusted for age, sex, duration, BMI, Cr, HbA1c, ALT, TC, TG, HDL and LDL. **(A)** TT4RI, thyrotrophic thyroxine resistance index, **(B)** TSHI, thyroid-stimulating hormone index, **(C)** TFQI, thyroid feedback quantile index, **(D)** PTFQI, parametric thyroid feedback quantile index, **(E)** FT3/FT4 ratio: free triiodothyronine to free thyroxine ratio,.

As illustrated in **Figure 2**, the receiver operating characteristics (ROC) curve analysis for the T2DM group indicated that TT4RI, TSHI, TFQI, PTFQI and FT3/FT4 values could serve as significant predictors of CHD (all *P* < 0.001), with areas under the curves (AUCs) of 0.732, 0.731, 0.732, 0.732 and 0.740, respectively. The central and peripheral TH sensitivity have the same efficacy in predicting the risk of CHD, but the area under the curve of FT3/FT4 is the largest, and its optimal cut-off value is 0.399.

### Mediation analysis using albumin

**Figure 3:**
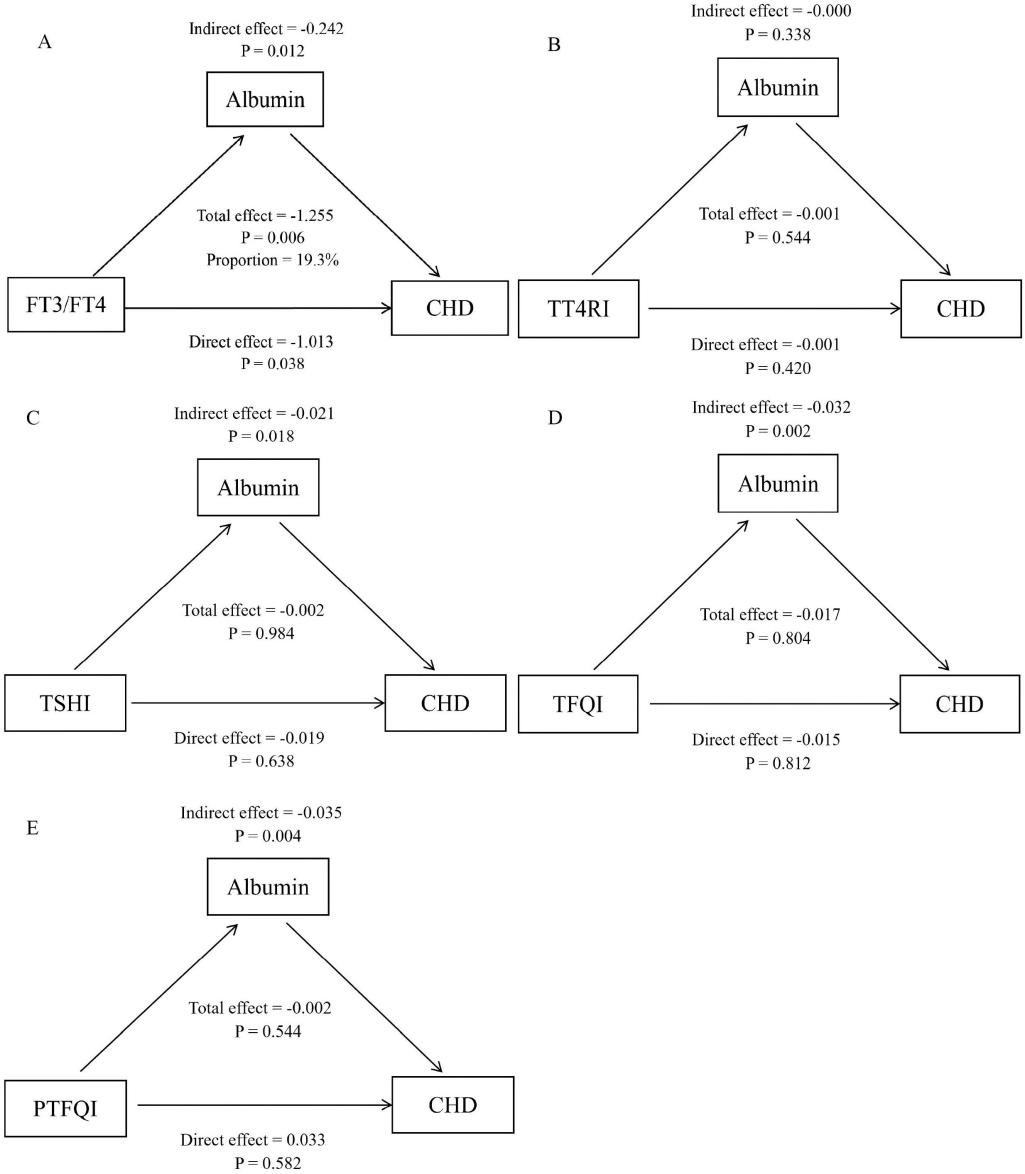
Mediation analyses of the association between TH sensitivity indices and CHD through albumin. CHD, coronary heart disease; TT4RI, thyrotrophic thyroxine resistance index; TSHI, thyroid-stimulating hormone index; TFQI, thyroid feedback quantile index; PTFQI, parametric thyroid feedback quantile index; FT3/FT4 ratio, free triiodothyronine to free thyroxine ratio.

**Figure 3** illustrates the potential mediating role of albumin in the association between TH sensitivity and CHD risk in patients with T2DM. The results showed that the FT3/FT4 ratio had a significant direct effect on CHD risk (β = -1.013, 95%CI (-1.923, -0.122), *P* = 0.038). Furthermore, albumin partially mediated this association, contributing a significant indirect effect (β = -0.242, 95%CI (-0.530 - 0.035), *P* = 0.012). The mediated proportion accounted for 19.3% of the total effect. Although the total effect of central TH sensitivity on the risk of CHD was not statistically significant, mediation analysis confirmed that albumin exerted a mediation effect in this relationship.

## Discussion

We performed a mediation analysis to quantify the direct and indirect effects of TH sensitivity on the risk of CHD among patients with T2DM. We found that impaired peripheral TH sensitivity was associated with an increased risk of CHD, whereas no significant association was observed for central TH sensitivity. Additionally, serum albumin potentially mediated the relationship between the FT3/FT4 ratio and CHD. To our knowledge, this is the first study to quantify the role of albumin in the association between TH sensitivity and CHD risk. These results suggest that peripheral TH sensitivity may affect cardiovascular health via its interplay with nutritional-inflammatory status.

Our findings demonstrate that peripheral TH sensitivity is negatively correlated with CHD in diabetic patients. This significant association persists even after adjusting for confounding factors such as age and sex. This is consistent with the findings reported by Neves JS et al. (27), who also identified that lower FT3/FT4 was associated with an increased 10-year risk of cardiovascular events. Wu YM et al. (28) also reported that the FT3/FT4 ratio is inversely associated with the presence and thickness of carotid plaques. The peripheral conversion of FT4 to FT3, primarily driven by type 1 (DIO1) and type 2 (DIO2) deiodinases, serves as a crucial indicator of metabolic health (29). It has been established that deiodinase activity can be directly inhibited under hyperglycemic conditions. Bapputty R et al. demonstrated that DIO2 levels in microvascular endothelial cells were significantly reduced in a high-glucose environment (30). Furthermore, the chronic inflammatory response induced by T2DM is a critical factor in impairing deiodinase function. Deiodinases are selenoenzymes that are highly sensitive to the cellular redox state (31). Pro-inflammatory cytokines, such as IL-6, induce the production of reactive oxygen species, which subsequently deplete intracellular reduced glutathione-an essential cofactor for deiodinase catalysis. This process leads to direct oxidative damage and functional impairment of the deiodinase enzymes (32). Furthermore, a clinical study demonstrated that serum insulin levels are significantly and negatively correlated with the FT3/FT4 ratio (33). TH promotes the production of nitric oxide in the vascular endothelium, thereby maintaining vasodilator function (34). When thyroid hormone levels decline or peripheral conversion is impaired, the activity of endothelial nitric oxide synthase decreases, leading to endothelial dysfunction and accelerating the progression of atherosclerosis and CHD (35).

We also found a stronger association between peripheral TH sensitivity and CHD in female with diabetes than male. Previous studies have demonstrated that although the prevalence of diabetes is similar between males and females, the detrimental impact of diabetes on the cardiovascular system is significantly greater in females than in males (36). Gong R et al. likewise demonstrated that the correlation between TH sensitivity indices (TFQI, PTFQI, and FT3/FT4 ratio) and carotid plaque were more pronounced among female individuals (37). This sex related difference in the relationship between TH sensitivity and CHD risk may provide crucial insights into the underlying endocrine mechanisms. Notably, this divergence could be attributed to hormonal variations, potentially involving estrogen, which is well known to modulate both thyroid function and immune responses (38, 39).

However, we found no significant association between central TH sensitivity and CHD. This divergence reflects the differential responses of various 5’-deiodinase isoforms to the pathological environment of diabetes (40). In contrast to peripheral tissues, central sensitivity is governed by DIO2 in the hypothalamus and pituitary (41, 42). Even amidst peripheral T3 deficiency, DIO2 maintains central hormone homeostasis by enhancing its own stability, thereby preserving the HPT axis feedback mechanism (43). Our findings emphasize that monitoring peripheral conversion indices offers greater predictive value for cardiovascular risk in diabetic patients than central indices alone. Furthermore, a previous cross-sectional study indicated that elevated TSHI, TT4RI, TFQI, and PTFQI were associated with an increased risk of carotid plaque (14). Conversely, Choe HJ et al. demonstrated an inverse association between PTFQI and cardiovascular risk among euthyroid Korean populations (44). These inconsistencies may stem from methodological differences, such as sample size, cohort demographics, and the established reference intervals for TH.

Furthermore, our results indicate that reduced albumin level potentially mediate the association between peripheral TH sensitivity and CHD risk. Mechanistically, this mediation can be explained through two primary pathways. As a critical transport protein for TH (45), hypoalbuminemia directly impairs hormone delivery to target tissues including the myocardium, thereby amplifying the impact of diminished peripheral sensitivity (46). Additionally, albumin is recognized for its potent anti-inflammatory and antioxidant properties (47), with circulating levels characteristically declining during systemic inflammation (16, 48). Consequently, reduced albumin levels in this cohort reflect an underlying state of chronic inflammation. This inflammatory state not only inhibits the binding of TH to their receptors (49) and the peripheral conversion of FT4 to FT3 (32), but also directly damages the vascular endothelium.

This is the premier study to assess TH sensitivity and CHD risk in the diabetic population while integrating serum albumin into the analytical framework. Nevertheless, several limitations should be acknowledged. Primarily, the cross-sectional design of this study precludes the establishment of causal relationships among TH sensitivity, albumin, and CHD. Further longitudinal or intervention studies are needed to confirm the relationship among these three factors, including approaches such as increasing albumin levels or enhancing TH sensitivity. Furthermore, our study cohort was relatively homogeneous and we failed to adjust for key confounding factors such as smoking, alcohol consumption, blood pressure, C-reactive protein and medication usage. These unmeasured residual confounding factors may potentially affect the robustness of the results. Consequently, the generalizability of our findings must be validated across diverse, multicenter populations.

## Conclusions

In conclusion, our study indicates that reduced peripheral TH sensitivity is associated with an elevated risk of CHD among T2DM patients, and highlights the potential mediating role of serum albumin. These findings offer valuable mechanistic insights into the crosstalk between thyroid homeostasis and cardiovascular pathogenesis. Ultimately, our results suggest that targeted interventions to optimize the nutritional and inflammatory profiles of patients with impaired TH sensitivity may represent a potential target for future investigation.

## Data Availability

The raw data supporting the conclusions of this article will be made available by the authors, without undue reservation.
