# Supplementary material for: Association between impaired peripheral thyroid hormone sensitivity and coronary heart disease in type 2 diabetes: the potential mediating role of albumin
Source: Front Endocrinol (Lausanne). 2026 Jun 17;17:1839913. doi: 10.3389/fendo.2026.1839913 (PMC13318788; doi:10.3389/fendo.2026.1839913)
Supplement: Supplementary file 1 [file DataSheet1.docx]

# 1 Supplementary Methods

## Inclusion and exclusion criteria of participants

The inclusion criteria were as follows: (1) age ≥ 18 years, (2) normal thyroid function, (3) availability of complete clinical data and (4) experiencing typical symptoms of diabetes combined with a random blood glucose level ≥ 11.1 mmol/L, a fasting blood glucose level ≥ 7.0mmol/L; or a blood glucose level ≥ 11.1 mmol/L after a 75 g oral glucose tolerance test. The diagnosis of CHD was established in accordance with the clinical diagnostic criteria of the International Society of Cardiology and the World Health Organization (WHO), and was confirmed via coronary angiography.

Patients were excluded from the study if they met any of the following criteria: (1) T1DM or diabetes secondary to other diseases, (2) history of thyroid disease or thyroid surgery, (3) previous treatment with medications that could alter TH concentrations (e.g., Iodine, amiodarone, phenytoin sodium), (4) a history of pituitary disease, (5) severe chronic liver disease, cancer, acute diabetes complications, and other severe system diseases, (6) pregnancy.

# 2 Supplementary Table and Figures

Table S1 Logistic regression analysis of TH sensitivity and the risk of CHD in diabetes according to sex

| Sex | Variables | Model 1 | | | Model 2 | | |
| --- | --- | --- | --- | --- | --- | --- | --- |
|  |  | OR | 95%CI | P | OR | 95%CI | P |
| Female | FT3/FT4 | 0.583 | 0.394 - 0.863 | 0.007 | 0.591 | 0.358 - 0.976 | 0.004 |
|  | TT4RI | 1.008 | 0.986 - 1.030 | 0.479 | 1.006 | 0.979 - 1.033 | 0.679 |
|  | TSHI | 1.233 | 0.655 - 2.321 | 0.516 | 1.227 | 0.553 - 2.724 | 0.615 |
|  | TFQI | 1.003 | 0.397 - 2.534 | 0.995 | 1.259 | 0.403 - 3.938 | 0.692 |
|  | PTFQI | 1.030 | 0.427 - 2.483 | 0.948 | 1.393 | 0.465 - 4.175 | 0.554 |
| Male | FT3/FT4 | 0.852 | 0.664 - 1.092 | 0.205 | 0.873 | 0.665 - 1.147 | 0.329 |
|  | TT4RI | 1.001 | 0.984 - 1.017 | 0.949 | 0.997 | 0.979 - 1.015 | 0.705 |
|  | TSHI | 0.873 | 0.564 - 1.351 | 0.542 | 0.875 | 0.545 - 1.46 | 0.582 |
|  | TFQI | 0.855 | 0.430 - 1.696 | 0.653 | 0.952 | 0.453 - 2.000 | 0.897 |
|  | PTFQI | 0.933 | 0.492 - 1.768 | 0.831 | 1.094 | 0.544 - 2.198 | 0.802 |

Notes: Model 1: crude model; Model 2: adjusted for age, sex, duration, BMI, Cr, HbA1c, ALT, TC, TG, HDL and LDL.

OR: Odd ratio, 95% CI: 95% confidence interval, FT3: free triiodothyronine, FT4: free thyroxine, TT4RI: thyrotrophic thyroxine resistance index, TSHI: thyroid-stimulating hormone index, TFQI: thyroid feedback quantile index, PTFQI: parametric thyroid feedback quantile index, FT3/FT4: free triiodothyronine to free thyroxine ratio.


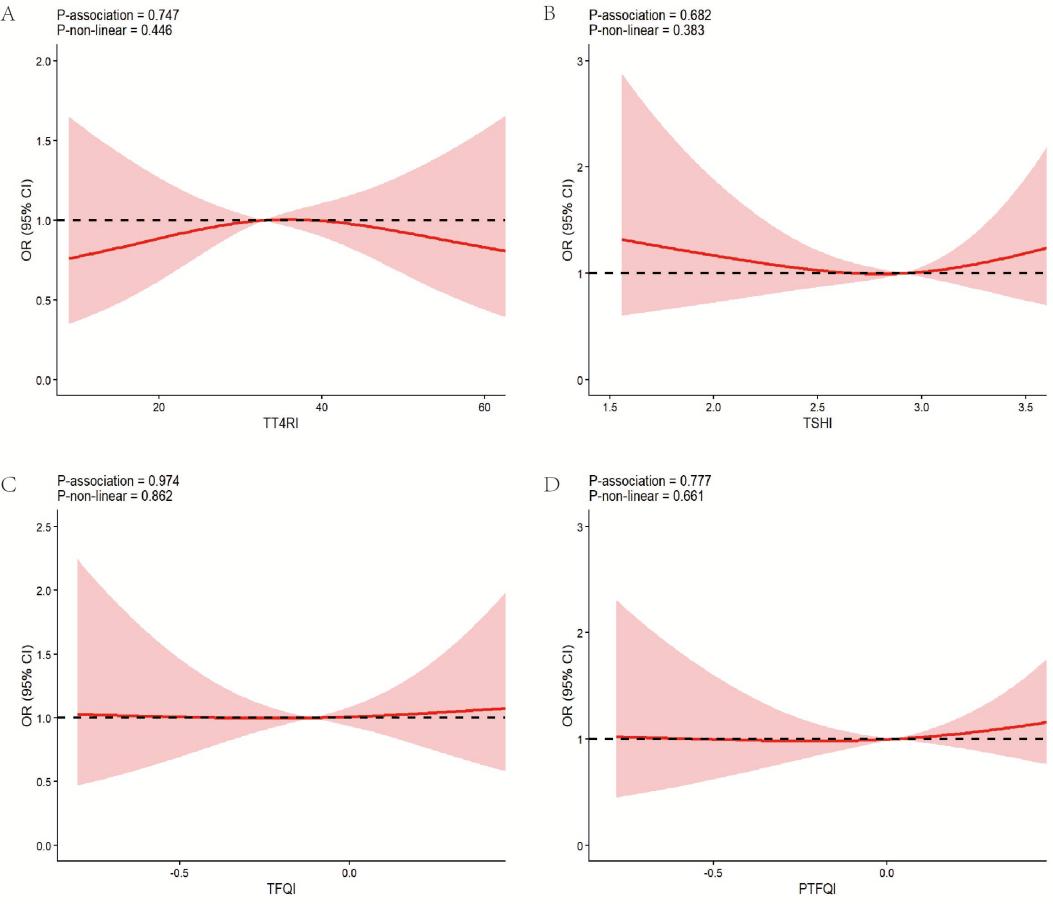


Figure. S1 Restricted cubic spline (RCS) analysis for the association between central TH sensitivity indices and type 2 diabetes with CHD after adjusting for age, sex, duration, BMI, Cr, HbA1c, ALT, TC, TG, HDL and LDL. TT4RI: thyrotrophic thyroxine resistance index, TSHI: thyroid-stimulating hormone index, TFQI: thyroid feedback quantile index, PTFQI: parametric thyroid feedback quantile index.
